# Inhibition Mechanism of Indoleamine 2, 3-Dioxygenase 1 (IDO1) by Amidoxime Derivatives and Its Revelation in Drug Design: Comparative Molecular Dynamics Simulations

**DOI:** 10.3389/fmolb.2019.00164

**Published:** 2020-01-28

**Authors:** Xinyu Liu, Yiwen Zhang, Huaichuan Duan, Qing Luo, Wei Liu, Li Liang, Hua Wan, Shan Chang, Jianping Hu, Hubing Shi

**Affiliations:** ^1^Laboratory of Tumor Targeted and Immune Therapy, Clinical Research Center for Breast, State Key Laboratory of Biotherapy, West China Hospital, Sichuan University and Collaborative Innovation Center for Biotherapy, Chengdu, China; ^2^Key Laboratory of Medicinal and Edible Plants Resources Development of Sichuan Education Department, College of Pharmacy and Biological Engineering, Sichuan Industrial Institute of Antibiotics, Chengdu University, Chengdu, China; ^3^College of Mathematics and Informatics, South China Agricultural University, Guangzhou, China; ^4^School of Electrical and Information Engineering, Institute of Bioinformatics and Medical Engineering, Jiangsu University of Technology, Changzhou, China

**Keywords:** indoleamine 2, 3-dioxygenase 1, epacadostat, molecular dynamic simulation, inhibition mechanism, revelation in drug design

## Abstract

For cancer treatment, in addition to the three standard therapies of surgery, chemotherapy, and radiotherapy, immunotherapy has become the fourth internationally-recognized alternative treatment. Indoleamine 2, 3-dioxygenase 1 (IDO1) catalyzes the conversion of tryptophan to kynurenine causing lysine depletion, which is an important target in the research and development of anticancer drugs. Epacadostat (INCB024360) is currently one of the most potent IDO1 inhibitors, nevertheless its inhibition mechanism still remains elusive. In this work, comparative molecular dynamics simulations were performed to reveal that the high inhibitory activity of INCB024360 mainly comes from two aspects: disturbing the ligand delivery tunnel and then preventing small molecules such as oxygen and water molecules from accessing the active site, as well as hindering the shuttle of substrate tryptophan with product kynurenine through the heme binding pocket. The scanning of key residues showed that L234 and R231 residues both were crucial to the catalytic activity of IDO1. With the association with INCB024360, L234 forms a stable hydrogen bond with G262, which significantly affects the spatial position of G262-A264 loop and then greatly disturbs the orderliness of ligand delivery tunnel. In addition, the cleavage of hydrogen bond between G380 and R231 increases the mobility of the GTGG conserved region, leading to the closure of the substrate tryptophan channel. This work provides new ideas for understanding action mechanism of amidoxime derivatives, improving its inhibitor activity and developing novel inhibitors of IDO1.

## Introduction

Cancer is the leading cause of death in pre-70s in 91 countries (McGuire, 2016), and annual cancer cases may increase to 22 million over the next two decades (Torre et al., 2015), increasing demand for anti-cancer drugs (Lin et al., 2018). Recent successes in cancer immunotherapy have generated significant interest in breaching immune suppressive mechanisms (Zeeberg Iversen et al., 2014), harnessing the body's immune system in the fight against cancer (Dance, 2017), after conventional operative treatment, chemotherapy, radiotherapy and targeted therapy (Pan et al., 2018). Immune checkpoint (Pardoll, 2012) inhibitors (ICIs), targeting programmed death protein 1 (PD-1)/programmed death-ligand 1 (PD-L1) (Brahmer et al., 2015; Reck et al., 2016; Antonia et al., 2017; Rittmeyer et al., 2017) and cytotoxic T-lymphocyte–associated protein 4 (CTLA-4), enhance T-cells mediated anti-tumor effect by blocking immune checkpoints and restoring immune cells' monitoring and killing function upon tumors (Abril-Rodriguez and Ribas, 2017). They were recently approved for a wide variety of cancer types and changed the landscape of therapy in cancer (Tang et al., 2018). With more immune targets identified, many novel immune therapies to enhance T-cell function have entered clinical trials (Komiya and Huang, 2018).

Similar to other immune checkpoints, indoleamine 2, 3-dioxygenase 1 (IDO1) is also suggested to be an important target for immunotherapeutic intervention (Zhai et al., 2015). It is the only rate-limiting enzyme outside the liver that catalyzes the oxidation of indole ring in the tryptophan molecule and metabolizes it along the kynurenine pathway (Yamazaki et al., 1985). Abnormal expression or overactivation of IDO1 tends to be associated with poor prognosis in a variety of human tumors (Godin-Ethier et al., 2011). IDO1 protects tumor cells from being cleared through three mechanisms, which are tryptophan depletion (Curti et al., 2007; Hill et al., 2007; Xie et al., 2008; Chang et al., 2011), products toxicity of tryptophan metabolites (Myint and Kim, 2014) and inducing regulatory T cell proliferation (Fallarino et al., 2006a,b; Colombo and Piconese, 2007; Kim et al., 2007). There are preclinical evidences that IDO1 inhibition can disrupt acquired immune tolerance, enhance tumor-specific killing (Jinushi et al., 2014), and significantly increase the immune response induced by various chemotherapeutic and immunotherapeutic agents (Zamanakou et al., 2007; Katz et al., 2008; Prendergast, 2008; Liu et al., 2010). In order to improve patient response rates and survival, interest exists in developing combination immunotherapies that target various immune evasion pathways (Zheng et al., 2006; Nagato and Celis, 2014; Schalper, 2014). In some clinical phase I/II models, epacadostat (INCB024360), one of IDO1 inhibitors, plus an ICI generally was well tolerated and produced relatively higher objective response rates (ORRs) in multiple advanced solid tumors (Hamid et al., 2017; Lara et al., 2017; Perez et al., 2017; Smith et al., 2017), primarily through the reactivation of tumor-infiltrating T cells and/or the decrease of tumor-resident immunosuppressive regulatory T cells (Spranger et al., 2014). However, in April 2018, the biotech company Incyte has made public the negative results of ECHO-301 (Garber, 2018) after a phase III trial in roughly 350 patients with unresectable or metastatic melanoma (in the trial, epacadostat/pembrolizumab combination and pembrolizumab alone were compared), which dampened enthusiasm with IDO1 inhibitors (Komiya and Huang, 2018). As the dominant viewpoint of the medical field, IDO1 is an important target for cancer drug development, but its inhibitory mechanism remains unknown. Some scientific experts believe that the drug, not the target might be the problem, as some IDO1 inhibitors bind the aryl hydrocarbon receptor (AHR) and thus suppress the immune system (Brochez et al., 2017). Anyway, understanding the inhibition mechanism of INCB024360 is the first step in rationally modifying the structure of IDO1 inhibitors to improve their activity and scope of application.

IDO1 is composed of two domains, among which the function of N-terminus domain is still unclear and may help favor the stability of the system, while the large C-terminus domain contains the heme active center performing the core functions of the enzyme. The C-terminus domain is rich in hydrophobic residues, which shows strictly complementary to the shape of the indole ring of the substrate, allowing the interaction of oxygen molecule (O_2_) with iron atom (Fe) in the first step of the reaction (Sugimoto et al., 2006). In the large domain, there is a ligand delivery tunnel for O_2_ and water molecules (H_2_O), which extends along the E and F α helix to the heme active center (Lewis-Ballester et al., 2017, 2018), as well as the 360-381 loop region controlling the shuttle of substrate/product in/out the catalytic site (Macchiarulo et al., 2007). The loop parallels with the heme plane before adding inhibitor, but moves to the small domain (i.e., N-terminus domain) after the association with IDO1/Trp/kynurenine, etc. Structural biology of IDO1 will be detailedly described in section 2.1.

Since the discovery of IDO1 in 1967, the development of IDO1 inhibitors has been mainly focused on the treatment of neurological disorders and neurodegenerative diseases (Cady and Sono, 1991; Ma et al., 1999). 1-Methyltryptophan (1-MT), which was structurally modified from the substrate tryptophan (Trp) as a template, was the first IDO1 inhibitor discovered (Yang et al., 2013; Li et al., 2014). After 2000, immunosuppressive effect of IDO1 was revealed, its inhibitors set off a new upsurge in anti-cancer drugs. In 2006, the crystal structure of human IDO1 complex with its inhibitor 4-phenylimidazole (4-PI) was determined, and the corresponding binding site was revealed (Sugimoto et al., 2006), introducing new ideas about the design of IDO1 inhibitors. Rational application of computer-aided drug design (CADD) has led to the discovery of a large number of effective IDO1 inhibitors with completely new skeletons. By exploring the binding mode of thiazole inhibitors with IDO1, Tojo et al. found another region (i.e., pocket B) that can bind to inhibitors in the vicinity of the active site (referred to as pocket A), which is important for exerting inhibitory functions (see figure S1A). Besides, they also suggested that the interactions with F226 and R231 are essential for potent IDO1 inhibitory activity using gene mutation analysis (Tojo et al., 2014). In 2017, Peng et al. proved that NLG919 analogs forms an extensive hydrogen bond network with IDO1, which greatly improved its inhibitory potency (Peng et al., 2016). Epacadostat (INCB024360) is the fastest-growing IDO1 small molecule inhibitor developed by Incyte (Yue et al., 2009; Yan et al., 2017). It has an IC_50_ value of 19 nM at cellular level and 70 nM at enzyme level (Liu et al., 2010). These impressive experimental data full establish INCB024360 as worthy of further study for the development of anti-tumor drugs (Shi et al., 2016; Beatty et al., 2017). The structure of INCB024360 was disclosed in 2015 and is now commercially available (Rohrig et al., 2015). In addition to high efficiency, INCB024360 is also a highly selective IDO1 inhibitor, whose effect was once highly anticipated in clinical research. The study on the inhibitory mechanism of INCB024360 is of great significance for the improvement of the inhibitory activity and structural modification of amidoxime derivatives.

After INCB024360 was picked out as a highly potent and selective IDO1 inhibitor by means of high-throughput screening (Yue et al., 2017), a series of biological experiments prove that amidoxime derivatives significantly suppress systemic tryptophan catabolism and the growth of IDO-expressing tumors (Smith et al., 2017). In addition, the recognition pattern of substrate Trp, the strategy of association enhancement and conformational transitions in indoleamine-2, 3-dioxygenase (IDO1) were systematically explored by molecular docking and spatial coarse graining simulations (Macchiarulo et al., 2007). Nevertheless, there are still several important scientific issues that remain unclear: What is the inhibition mechanism of the highly active INCB024360? How does the conformation of IDO1 change after the association with INCB024360? What are the details of the identification and action between INCB024360 and IDO1? Accurate answers to these questions are very important for the development of novel INCB024360 derivative inhibitors and will help reveal potential remodeling sites in IDO1. In this paper, the overall stability and conformational change of IDO1 complex with INCB024360 both were analyzed by comparative molecular dynamics simulations, and then the action mechanism of the inhibitor was investigated using free energy landscape (FEL), principal component analysis (PCA) and key residues scanning methods. Finally, the key residues that play an important role in identification and catalysis were revealed through the analyses of hydrogen bond, structural superimposition, as well as distance and angle monitoring, and potential modification sites of inhibitors were proposed. This study not only clarifies the inhibition mechanism of INCB024360, having theoretical significance, but also is helpful to obtain highly active amidoxime derivatives, showing important application values.

## Results and Discussion

### Structural Biology of IDO1

By July 2019, total 35 crystal structures of IDO1 were recorded in the RCSB Protein Database (www.rcsb.org). Table S1 lists specific information on these structures. From Table S1, we can see that they are all obtained by X-ray diffraction method. All of them are homo sapiens, and ligands that bind to IDO1 include its substrate Trp, inhibitor, effector and other small molecules helping to stabilize protein structures. In order to study the significance of certain amino acids or obtain a high-resolution crystal structure, some structures also contain one or two mutation sites.

**Figure 1:**
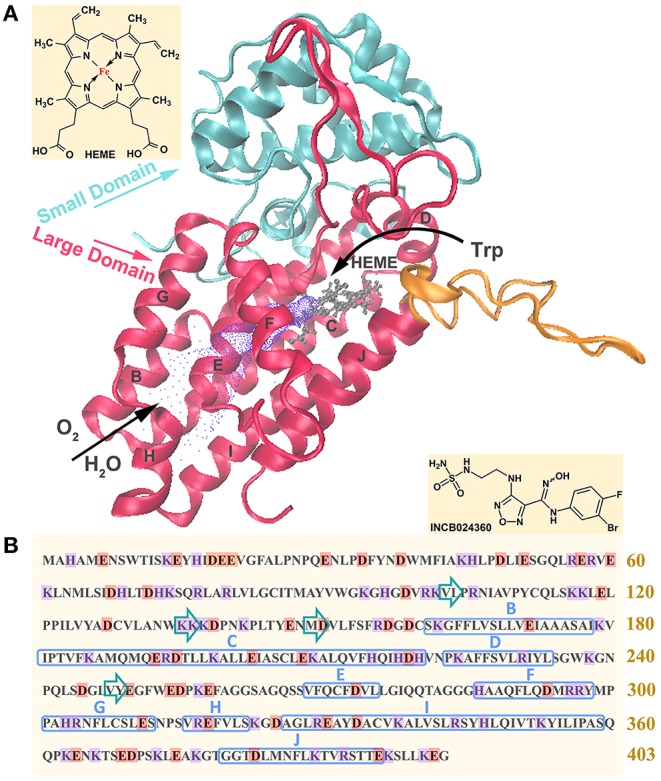
Representation of tertiary **(A)** and primary **(B)** structures of IDO1. In figure **(A)**, blue and red represent the small domain and large domain, respectively; ligand delivery tunnel is colored in purple, where O_2_ and H_2_O pass through to heme active center; the substrate/product shuttle channel is colored in orange, where Trp passes in and out the catalytic site; the active site containing heme is colored in gray. In figure **(B)**, acidic amino acids are marked in orange and basic amino acids are marked in purple; α helixes and β sheets are respectively marked with blue rectangle and green arrows.

Figure 1 shows its primary sequence and tertiary structure, as well as the molecular structure of inhibitor INCB024360. IDO1 functions as a monomer containing 403 amino acids, composed of a small N-terminal domain, a large C-terminal domain (containing 9 α-helices, where specific α helixes are marked in Figure 1B), and a cofactor heme molecule (van der Goot et al., 2012). Large domain is rich in functions, including catalytic site, ligand delivery tunnel and Trp/product shuttle channel, which are, respectively colored in gray, purple and orange in Figure 1A. While small domain may only serve as structural support. In terms of catalytic processes, small molecules such as oxygen molecules first arrive at the active site (around L201-E250 segment) through the ligand delivery tunnel (around G251-P300 segment), and then bind with Fe in heme to induce the next reaction (Hu et al., 2016). Simultaneously, the substrate Trp enters the active site, passing through the substrate/product shuttle channel (around Q360-G381 segment), and then plays its catalytic role (Macchiarulo et al., 2007; Lewis-Ballester et al., 2018).

### Structural Completion and Rationality Verification

In order to explore the inhibition mechanism of INCB024360 against IDO1 and the revelation in drug design, the most appropriate research system was selected through a carefully weighing of IDO1 structures. Firstly, according to the resolution, organism and ligands type of crystal structure, the first five IDO1 crystal structures with no mutation were selected (i.e., PDB IDs: 2D0T, 5WHR, 5EK3, 5WN8, and 6F0A). Figure S1A records details of screening results. By stacking them and using 2D0T as the initial conformation, the root-mean-square deviations (RMSDs) of other IDO1 conformations with 2D0T were calculated to compare differences in different crystal structures. RMSDs of the four systems are all <0.4 Å, indicating that there are slight differences in conformations. Therefore, the crystal structure containing INCB024360 was selected for subsequent analysis (PDB ID: 5WN8). We also analyzed the binding pattern between different types of inhibitors and IDO1, selecting 4-phenylimidazole, NLG919 analog and INCB024360 as representative small molecules. According to the crystal structure, they all have the similar binding pattern, in which the stable polar bond is formed by N or O with Fe in heme, the larger aromatic group is oriented toward the hydrophobic pocket A (composed of Y126, C129, V130, F163, F164, L234), and the long-chain hydrocarbon moiety faces the hydrophobic pocket B (composed of F226, A264, R231, I354, L384). The similarity of binding modes between different IDO1 inhibitors indicates that the inhibition mechanism of amidoxime derivatives and its revelation in drug design both have certain guiding significance for developing novel IDO1 inhibitors.

Considering the incompletion of 5WN8 PDB structure, it is necessary to complete the system and prove its rationality. we first compared it with the IDO1 primary amino acid sequence using EBI's multiple sequence alignment of protein sequences function (Madeira et al., 2019), and then found that amino acids M1-K13 and Q361-G378 of 5WN8 are missing (see Figure S1B). Residue completion was conducted by SWISS-MODEL server (swissmodel.expasy.org). After structural completion, the structural rationality was evaluated with the QMEAN Z-score and Ramachandran distribution. The QMEAN Z-score provides an estimate of the “degree of nativeness” for the structural features observed in the model on a global scale. It indicates whether the QMEAN score (Benkert et al., 2011) of the model is comparable to what one would expect from experimental structures of similar size. QMEAN Z-scores around zero indicate a good agreement between the model structure and experimental structures of similar size. Scores of −4.0 or below is an indication of models with low quality. According to Figure S1C, the lower absolute quality score indicates the IDO1 model structure is reasonable and reliable. The Ramachandran plot was able to evaluate the torsion degree of C_α_-C and C_α_-N bonds within peptide bonds of proteins and indicated the allowable and prohibitive conformations of residues. By observation, the dihedral angle distributions of all residues in IDO1 model were reasonable (see Figure S1D).

### Overall Dynamic Characteristics of the Trajectories

Two 100 ns comparative molecular dynamics simulations were performed for the IDO1 and IDO1-INCB024360 systems, respectively named IDO and IDO-BBJ. The potential energy of the two systems approached equilibrium after 2.50 ns, which shows the reasonability of MD simulations. The radiuses of gyration are both around 22.0 Å, indicating that the volume of IDO1 has not significantly changed (see Figure S2A). Then, we observed C_α_ atomic RMSD changes of IDO and IDO-BBJ systems, respectively tending to be stable with the values of 2.56 ± 0.61 Å and 2.24 ± 0.39 Å after 10 ns. The RMSD values of IDO-BBJ is relatively lower, which indicates that the inhibitor stabilized IDO1 protein to some extent (see Figure S2B). Figure S2C shows the distribution of root mean square fluctuation (RMSF) of C_α_ atoms in IDO and IDO-BBJ systems. The RMSF distributions of the two systems are similar on the whole, with some local differences: the pocket region (L200-R231) undergoes a significant conformational change; the flexibility of residues L103, L234, and I354 declines after the association with the inhibitor. The flexibility change in these regions may be related to the catalytic activity of IDO1, which will be further explored in detail. In Figure S2D, there is a high positive correlation between the calculated B-factor values and experimental ones in IDO system (R = 0.513, *N* = 389), which suggests that the obtained MD trajectories and subsequent conformational analysis both are reliable.

### Time-Dependent Conformational Cluster

**Figure 2:**
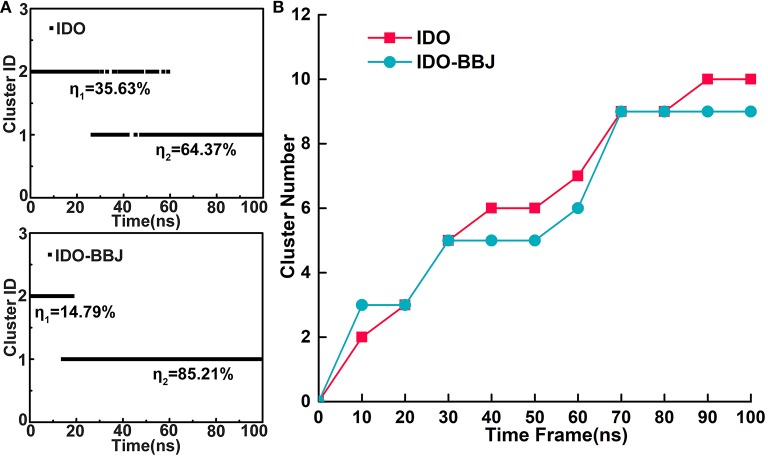
Time-dependent conformational cluster analysis. Overview of clusters with RMSD threshold of 2.4 Å **(A)**. Cluster variations over simulation time in the IDO and IDO-BBJ systems **(B)**.

To discuss details of conformational changes of IDO1 before and after binding inhibitor more accurately, time-dependent conformational cluster analyses were performed. Firstly, RMSD is set to 2.4 Å as a threshold to observe rough clusters (see Figure 2A). In IDO system, conformations are divided into two clusters throughout the simulation, where 35.63% conformations are classified as cluster 1 and the remaining 64.37% as cluster 2. Besides, it can also be seen that the conformations at adjacent time may be grouped into different clusters, showing relative obvious conformation transition characteristics. In contrast, conformations of IDO-BBJ system are also divided into two clusters, in which the larger cluster accounts for 85.21% and the corresponding conformational transition tends to be less. To better understand the effects of inhibitor on conformational change of the protein, the threshold value of RMSD is reset as 1.5Å to observe total clusters over simulation time. Figure 2B shows the time-dependent clustering results of the two systems. In the IDO-BBJ system, cluster number reaches steady state at 70 ns, however, that of IDO system is still growing till 80 ns. This phenomenon confirms the conjecture above-mentioned that the binding of INCB024360 helps stabilize IDO1 protein. Moreover, the convergence of time-dependent conformation clusters also suggests the sufficiency of conformational sampling, which is very necessary for subsequent molecular recognition and inhibitory mechanism discussion.

### Global Conformational Changes

**Figure 3:**
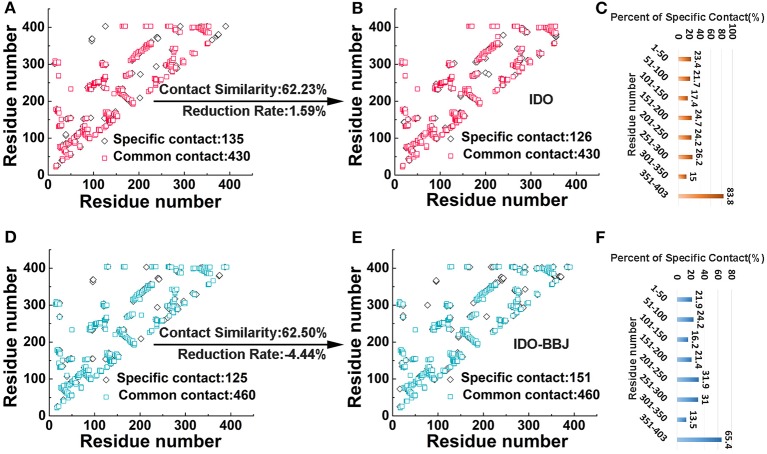
Comparative analyses of residue contacts in the IDO (colored in red and orange) and IDO-BBJ systems (showed in light blue and blue): residue contacts maps **(A,B,D,E)**, and percent of specific residue contacts in the two investigated systems **(C,F)**.

Residue contact map is an effective method used to describe the conformational change and motional status of proteins (Feig et al., 2004). When the distance between two residues of a protein is <4.5 Å (Hu et al., 2016), a residue contact occurs. In the IDO (Figures 3A,B) and IDO-BBJ (Figures 3D,E) systems, we investigated the difference of residue contacts between the first (0 ns) and the last (100 ns) snapshot. The number of residue contacts in the two initial conformation is 565 and 585, respectively. The final residue contacts were reduced to 556 in IDO system, however, increased to 611 in IDO-BBJ system. There are 430/460 common residue contacts in the two structures, while that of specific residue contacts is 135/126 and 125/151, respectively (see Figures 3A,D).

Herein, two parameters (i.e., contact similarity and reduction rate) were defined to demonstrate the conservativeness of residue contacts, as well as the extent of expansion and relaxation of MD systems (Kwak et al., 2017; Sun et al., 2018). Contact similarity is calculated by common contacts divided by all contacts that contain common and specific contact in initial and final structures. Reduction rate is computed by dividing the difference of specific contacts between the first and the last conformations by total contacts of the structure at 0 ns. The contact similarity and reduction rate of IDO and IDO-BBJ systems are 62.23/62.50% and 1.59%/−4.44%, respectively. According to the contact similarity value, both systems show strong conservation. The reduction rate of IDO-BBJ complex indicates that it is getting more compact after MD simulation.

In order to explore the reason why reduction rate changes so much, the peptides with large changes in the number of specific residue contacts need to be identified. For the convenience of analysis, IDO1 protein was divided into eight peptides with 50 amino acids as intervals, and then the ratio of the number of specific residue contacts in each peptide to the total number of residue contacts was calculated (see Figures 3C,F). By comparing the percent of specific contact between IDO and IDO-BBJ, it is found that the contact values of residues 201–250 and 251–300 of IDO1 are surprisingly higher after binding the inhibitor. In fact, the segments of 201–250 and 251–300 just constitute the binding pocket and active site access tunnel (Lewis-Ballester et al., 2017), respectively. In addition, regardless of IDO1 binding with the inhibitor or not, the specific contact value of the 351–403 segment is also very high. To explore the reasons behind these phenomena, the conformational changes of these three parts in two systems will be discussed in detail below.

**Figure 4:**
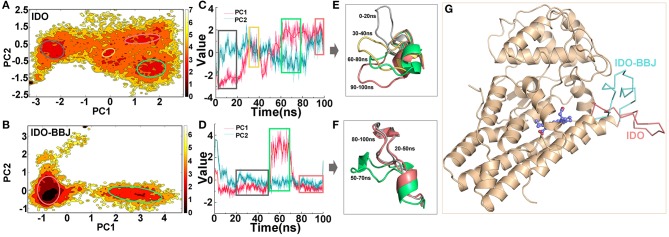
Free energy surface analyses of Q360-G381 loop in IDO1. Free energy landscapes of IDO and IDO-BBJ systems **(A,B)**. Conformational fluctuations of the first principal component (PC1) and the second principal component (PC2) vs. simulation time in the two systems, where the period corresponding to the lower energy conformation can be determined **(C,D)**. Conformational change of Q360-G381 loop in different time periods **(E,F)**. The open and close structures of Q360-G381 loop, which are colored in red and blue, respectively **(G)**.

Figures 4A,B display the free energy surfaces of IDO and IDO-BBJ systems at 300 K, respectively. The dark areas indicate higher conformation distribution probability, representing the most stable conformation states in MD simulations. As can be seen, the two systems have four and two representative conformations with low free energy, respectively. From Figures 4C,D, both PC1 and PC2 show non-Gaussian distributions in the two systems, especially PC1, which means that two principal components are representation of functional slow motions, thus principal component analyses (PCA) are reasonable and adoptable. As shown in Figures 4A,C, the four relative independent low free energy regions of the IDO system are, respectively corresponding to 0–20, 30–40, 60–80 and 90–100 ns. IDO-BBJ has only two low free energy regions, indicating that molecular motion of IDO1-INCB024360 complexes is less than that of IDO1 monomer, which is consistent with previous cluster analysis. Besides, the two regions correspond to the three time periods, which are 20–50, 50–70, and 80–100 ns (see Figure 4D). In the residue segment 351-403 with largest change in specific contacts, the flexible Q360-G381 loop controls the shuttle of substrates and products (Macchiarulo et al., 2007). Figures 4E,F give the representative Q360-G381 loop structures in the different time periods. It can be seen that the loop is relatively flexible. In IDO system, the Q360-G381 loop gradually stretched outward (i.e., open conformation), while in IDO-BBJ system, the loop keeps stable near the N-terminal small domain (i.e., close conformation). Figure 4G shows the open (in red) and close (in blue) structures of substrate/ product shuttle channel.

In fact, by deeply understanding the opening and closing mechanism of Q360-G381 loop, the attempt to design novel compounds to prevent Trp substrates from entering the active site could serve as an idea for the development of IDO1 inhibitors.

### Comparative Pore Profiles

**Figure 5:**
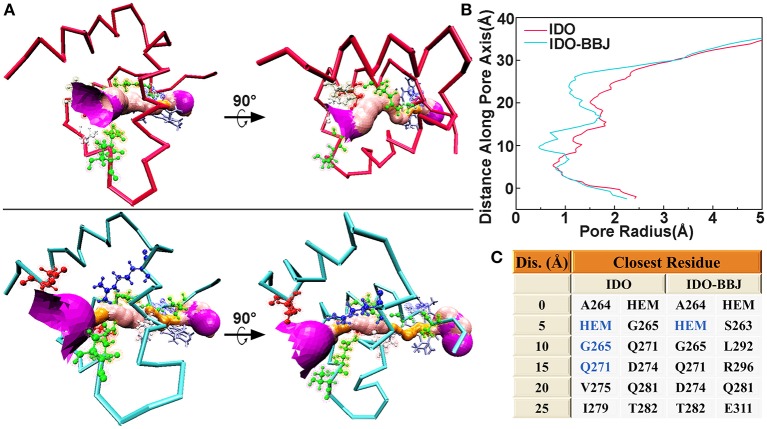
Hole analyses of G251-P300 segment in the IDO and IDO-BBJ systems. Tunnel visualizations **(A)**. The radius of gold pore is too small for accommodating water molecules, while the pink and purple pores, respectively allow a single and over two water molecules to pass through. Pore radius profiles vs. position along pore axis **(B)**. The closest residues to tunnels at different positions along pore axis **(C)**.

HOLE is a program for obtaining molecular characteristics of ion channels or active pocket through the visualization of their pore dimensions (Smart et al., 1996). The ligand delivery tunnel of IDO1, which is around residues G251-P300 and between E/F α helixes, was first identified as water tunnel. In 2018, Ballester et al. confirmed that the tunnel is a conduit delivering O_2_ into the heme-binding active site (Lewis-Ballester et al., 2018). Figure 5 shows ligand delivery tunnel of the IDO and IDO-BBJ systems. It can be seen that the tunnel in the IDO system is relatively wide and short, while that in the IDO-BBJ system becomes narrow and long. In particular, the position around 30 Å from pore center is too narrow to allow water molecules to pass through, which means that the passage efficiency of various ligands, such as H_2_O and O_2_, declines. Figure 5B shows changes of pore radius along the pore axis. We can more intuitively observe that, compared with IDO system, the pore channel of IDO-BBJ system becomes longer and the pore radius decreases from 0.8 to 0.5 Å. Figure 5C shows the closest residue to tunnel in the two systems at different distance along pore axis. As for the adjacent closest residues, heme, G265 and Q271 appear repeatedly in IDO, while only heme still remains in IDO-BBJ.

To sum up, we can draw a conclusion that the binding of INCB024360 to IDO1 significantly affects the shape of active pockets access tunnel and then inhibits IDO1's catalytic function by blocking the flow of oxygen molecules.

### Binding Free Energy Calculation

**Figure 6:**
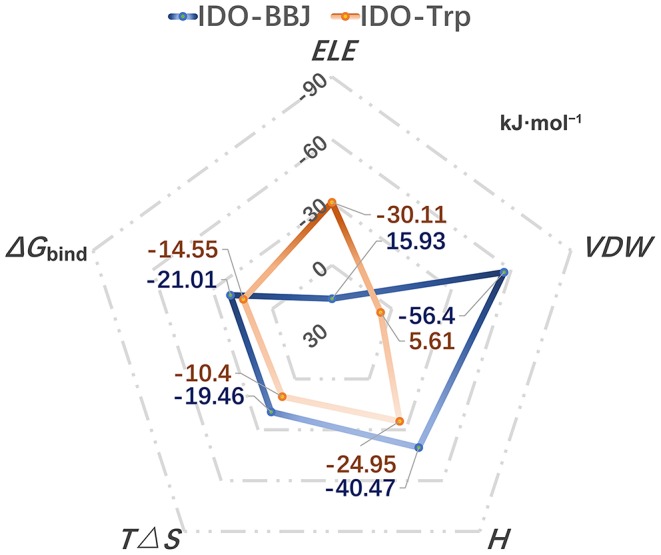
Calculated binding free energies in the IDO-Trp and IDO-BBJ systems, as well as the main driving force for the formation of complexes.

Binding free energy is the final criterion to effectively evaluate the strong or weak binding capacity between receptor and ligand. In order to compare the binding capacity of INCB024360 and Trp with IDO1, 100 ns MD simulation of IDO-Trp system was performed, and then the binding free energies of IDO-Trp and IDO-BBJ systems were, respectively predicted using Molecular Mechanics/Poisson Born Surface Area (MM/PBSA) method. Figure 6 shows the calculated binding free energies in the IDO-Trp and IDO-BBJ systems and calculation details are listed in Table S2. In IDO-BBJ, the enthalpy (*H*) and entropy changes (*T*Δ*S*) are −40.47 and −19.46 kJ·mol^−1^, respectively. Thus, the calculated absolute binding free energy (Δ*G*_bind_) is −21.01 kJ·mol^−1^, which is well-consistent with the reported experimental value (−23.6 kJ·mol^−1^) (Yue et al., 2017). As for the IDO-Trp system, the Δ*G*_bind_ is relative higher and maintains at −14.55 kJ·mol^−1^, indicating IDO1 prefers to bind with INCB024360 rather than substrate Trp.

Binding free energy can be decomposed into polar (*ELE*_IN_ + *ELE*_PB_ = *ELE*) and nonpolar (*VDW*_IN_ + *VDW*_PB_ = *VDW*) parts according to the MM/PBSA method. The two core values in the IDO-BBJ and IDO-Trp systems are 15.93/ −56.4 and −30.11/ 5.61 kJ·mol^−1^, respectively. It is found that the non-polar interactions can promote the formation of IDO-INCB024360, being the important driving force for their binding. Nevertheless, polar interactions are the main driving force of IDO-Trp. In a word, INCB024360 shows higher IDO1 binding capacity than endogenous substrate Trp, which was dominated by hydrophobic interactions.

### Key Residues Scanning

Energy decomposition was utilized to identify key residues facilitating the formation of IDO1-INCB024360 complex. Sorted by *E*_TOT_ value, Table 1 lists the top ten residues that contribute the most to the association of INCB024360 with IDO1. Eight of them are pocket-constituting residues (i.e., R231, L234, A264, Y126, F163, L384, I354, and F226), in which R231 seems to be the most critical one. As mentioned earlier, the inhibitor binding results in a significant decrease in L234 flexibility (see section Free Energy Landscape and Principal Component Analysis). Besides, in the IDO1-INCB024360 system with relatively lower binding energy, G262-S263 flexible loop has a dominant orientation outside pocket A. The conformational problems involved in all the key residues will be discussed in detail next.

**Table 1:** The top 10 key residues of IDO1 contributing to the association with INCB024360.

**Residues**	***E*_**VDW**_**	***E*_**ELE**_**	***E*_**GB**_**	***E*_**GBSUR**_**	***E*_**TOT**_**
R231	−1.63 ± 0.84	−11.59 ± 2.9	8.03 ± 1.45	−0.16 ± 0.03	−5.35 ± 1.64
L234	−2.4 ± 0.28	−1.31 ± 0.32	1.46 ± 0.35	−0.12 ± 0.02	−2.37 ± 0.31
S263	−1.85 ± 0.28	0.18 ± 0.49	0.01 ± 0.24	−0.13 ± 0.03	−1.78 ± 0.35
A264	−1.44 ± 0.18	−0.13 ± 0.32	0.28 ± 0.22	−0.17 ± 0.02	−1.46 ± 0.23
Y126	−1.25 ± 0.17	0 ± 0.09	0.22 ± 0.08	−0.06 ± 0.01	−1.09 ± 0.17
F163	−1.4 ± 0.31	−0.44 ± 0.14	0.92 ± 0.18	−0.17 ± 0.03	−1.08 ± 0.25
L384	−0.72 ± 0.26	−0.26 ± 0.24	0.29 ± 0.12	−0.14 ± 0.03	−0.82 ± 0.22
I354	−0.79 ± 0.19	0.01 ± 0.04	0.05 ± 0.03	−0.05 ± 0.03	−0.78 ± 0.19
G262	−1.47 ± 0.34	−2.93 ± 1.25	3.84 ± 0.49	−0.15 ± 0.03	−0.71 ± 0.75
F226	−0.93 ± 0.27	−0.14 ± 0.07	0.49 ± 0.12	−0.09 ± 0.02	−0.67 ± 0.33

**Figure 7:**
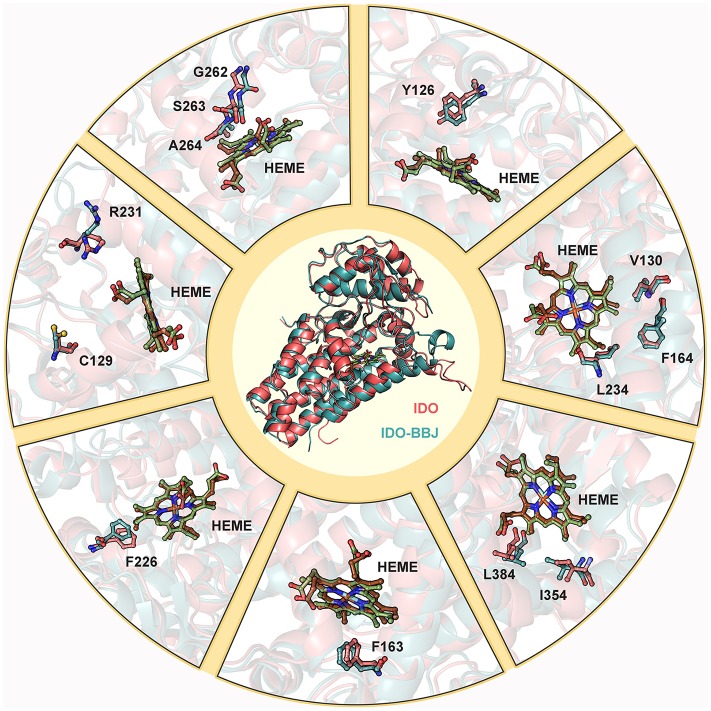
Superimposition of key residues in IDO and IDO-BBJ systems. Hemes in IDO and IDO-BBJ are shown in green and brown, respectively.

Figure 7 shows superimposition of key residues in the IDO (stick mode in red) and IDO-BBJ (stick mode in blue) systems after 100 ns MD simulations, respectively. The key residues here consist of those involved in the composition of pocket A and B, as well as those having important contributions to molecular recognition. Combined with Figure 7 and experimental information (Chauhan et al., 2008; Austin et al., 2013; Nienhaus et al., 2017; Tomek et al., 2017), the conformational changes of these key residues can be classified into the following four cases: (1) the conformation of C129, R231, G262, S263, and A264 have changed dramatically, and the last three gradually approach heme; (2) some residues still remain stable, such as V130, F164, and L234; (3) in order to better bind to INCB024360 containing multiple aromatic rings, some of aromatic residues—Y126, F163, and F226—undergo adaptive conformational changes; (4) induced by the long hydrocarbon chain tail of INCB024360, I354 and L384 both move closely to each other.

### Hydrogen Bond Analysis

Hydrogen bonds have specificity and closely relate to protein function, which plays a crucial role in maintaining structural stability and identifying ligands. Table 2 lists the hydrogen bonds around pockets in IDO and IDO-BBJ systems. Here, H-bonds were determined by geometric criterion of the distance between hydrogen atoms and acceptor atoms <3.5 Å and the angle of Acceptor-H-Donor larger than 135° (Thorson et al., 1995; Hu et al., 2008; Xie et al., 2019).

**Table 2:** The hydrogen bonds around pockets of IDO and IDO–BBJ systems during MD simulation.

**Systems**	**H–bonds**	**Distance (Å)**	**Angle (^**°**^)**	**Frequency (%)**
IDO	G171–OE1/Y126–HH	2.70	166.39	93
	V248–O/L103–H	2.87	163.43	78
	**G380–O/R231–HH22**	**2.82**	**155.44**	**36**
	G171–OE1/Y126–HH	2.73	165.74	83
IDO–BBJ	V248–O/L103–H	2.87	164.23	79
	**L234–O/G262–H**	**2.86**	**156.08**	**58**
	**INCB024360–N25/R231–HH21**	**2.89**	**159.36**	**29**

As shown in Table 2, there are two stable hydrogen bonds—G171-OE1/Y126-HH and V248-O/L103-H—in both IDO and IDO-BBJ systems, which play an important role in maintaining the stability of IDO1. In the IDO system, R231 forms a hydrogen bond with G380. Nevertheless, in the IDO-BBJ system, this interaction is broken and replaced by the hydrogen bond between R231 and INCB024360 (shown in bold). It is worth noting that G380 lies in the “GTGG” motif of the I-J Loop region (see Figure 1), which is fully conserved in IDO1 and TDO (tryptophan 2, 3-dioxygenase) families of enzymes (Lewis-Ballester et al., 2017). The resetting of G380-R231 hydrogen bond may be the main factor for the conformational change of Q360-G381 loop. The interactions of INCB024360 with R231 destroys the original open state of Q360-G381 loop, thereby closing the channel and preventing the substrate Trp from entering the active site. In IDO-BBJ system, a new L234-G262 hydrogen bond appears (shown in bold), which will be discussed in detail below.

**Figure 8:**
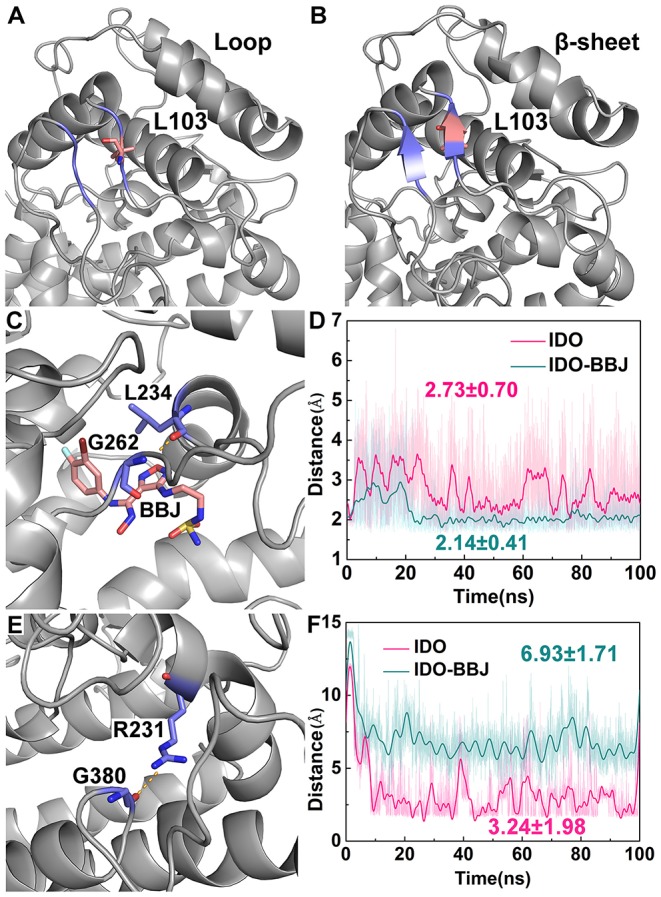
Distance analyses of key residues. Overview of residues around L103 in IDO1 monomer **(A)** and IDO-INCB024360 complex **(B)**; relative positions of INCB024360, G262 and L234 **(C)**, as well as distance variation of G262 with L234 over simulation time **(D)**; relative positions of G380 and R231 **(E)**, as well as distance variation of G380 with R231 over simulation time **(F)** in the IDO and IDO-BBJ systems.

Figure 8 shows the distance variation of some key hydrogen bond over simulation time. L103 is located in the link of N- and C-domains, and the disordered loop around it turns into an ordered β-sheet after 100 ns MD simulation, which may favor keeping stable and structurally tight between of N- and C- domains of IDO1 (see Figures 8A,B). According to distance analysis between G262 and L234, it is found that L234 is reduced in flexibility and remains stable in position after binding to inhibitor. Specifically, G262-A264 loop gradually approaches to L234 till the hydrogen bond of G262 with L234 is formed (see Figures 8C,D). In fact, G262-A264 loop is directly connected to the E α helix, which participates in regulating the entry of reactants to heme active site. It is speculated that INCB024360 first shows stronger IDO1 binding ability than endogenous substrate Trp, then attracts and stabilizes the G262-A264 loop, resulting in disorder of the tunnel, which substantially prevents small molecules such as O_2_ from entering the active site. Moreover, the hydrogen bond distance between G380 and R231 maintains around 3.24 Å in IDO1 monomer, while increasing to 6.93 Å in IDO1-INCB024360 complex (see Figures 8E,F).

Presumably, the cleavage of hydrogen bond between R231 and G380 is related to an increase in the mobility of Q360-G381 loop, eventually leads to the closure the channel of substrate Trp.

**Figure 9:**
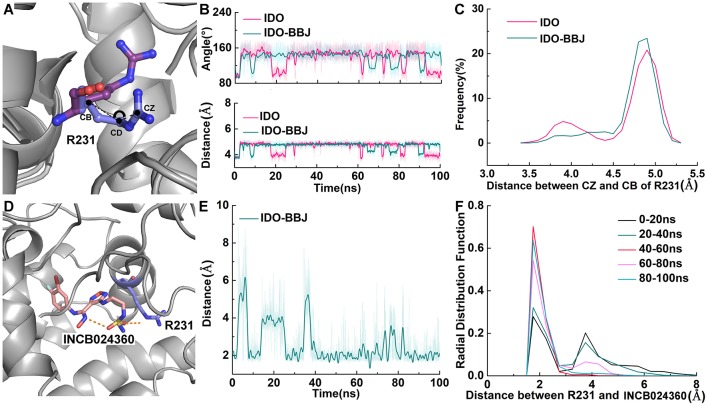
Detailed conformational analyses of R231. Angle, distance and frequency analysis of R231 in IDO and IDO-BBJ systems **(A–C)**. Distance and RDF analysis between INCB024360 and R231 **(D–F)**.

Free energy decomposition and hydrogen bond analysis both show that R231 may be the most important residue for INCB024360 to exerting its inhibitory activity. Figure 9 shows the detailed conformational analyses of R231 with INCB024360. According to calculated angle of CD and distance between CB and CZ (see Figure 9B), R231 possesses two relatively stable conformations: (1) the side chain of R231 is more folded (colored in blue, see Figure 9A); (2) the side chain is more stretched (colored in purple, see Figure 9A). Quantum chemical calculations show that the stretched conformation is more stable than the folded one (data not shown), and R231 in IDO-BBJ system is more likely to adopt the former conformation, which can be seen more visually in frequency analysis (see Figure 9C).

Obviously, full consideration of the stability of R231 stretched conformation will be beneficial to design IDO1 inhibitors with higher inhibition efficiency. Radial distribution function (RDF) is an effective strategy to observe the interactions between protein and its substrates, such as protein, DNA or small molecules (Ul Haq et al., 2017). From Figures 9D–F, the fluctuation amplitude and absolute distance between R231 and INCB024360 gradually decrease with simulation time. After 40 ns, a relatively stable hydrogen bond is formed with distance around 1.9 Å and frequency around 0.6. Based on this observation, changing the charge characteristics of INCB024360 will help enhance the electrostatic/ hydrogen bond interactions with R231, which may be one of the optimization ideas worth trying.

### Possible Inhibition Mechanism of INCB024360

**Figure 10:**
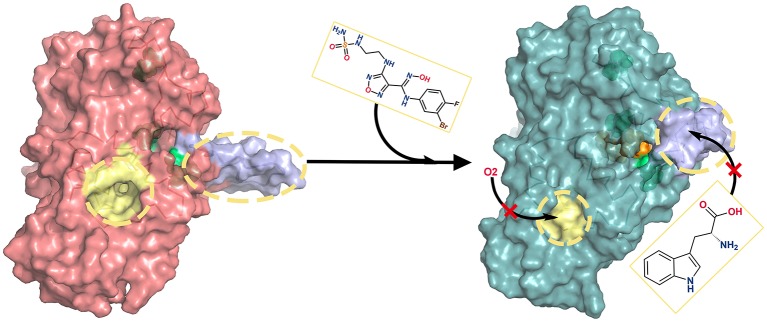
Inhibition mechanisms of INCB024360. In the absence of INCB024360, the ligand delivery tunnel (colored in yellow) of IDO1 is spacious, and the substrate/product shuttle channel (colored in purple) is opened. After binding inhibitor, ligand delivery tunnel becomes so narrow that small molecules (such as O_2_) cannot enter smoothly. Simultaneously, the substrate/product shuttle channel turns off and prevents substrate Trp from arriving in the IDO1 catalysis site.

Through the analyses of molecular structure superimposition, residue contact analysis and comparative MD simulations, the possible inhibition mechanism of amidoxime derivatives (i.e., INCB024360) is proposed (see Figure 10). First, INCB024360 approaches to the active site of IDO1 and causes the adaptive conformational changes of the pocket, which is more conducive to molecular recognition of IDO1-INCB024360. Second, the tail of INCB024360 forms a hydrogen bond with the structurally flexible R231 to break the original stable state of loop Q360-G381, which increases its mobility and then leads to the closure of the small molecule shuttle channel on the other side of active site. Besides, the formation of a stable hydrogen bond between INCB024360 and L234 reduces the flexible of this residue, which stabilizes the conformation of G262-A264 loop, resulting in a narrow and disordered tunnel between E and F α helixes and ultimately blocking the entry of small molecules such as O_2_.

In conclusion, the association with INCB024360 weakens the passage efficiency of both ligand delivery tunnel and substrate shuttle channel, suppresses the IDO1 enzyme activity in cancer cells, and further activates the immune killing function of human body.

### Revelation in Drug Design

In 2006, Sugimoto et al. demonstrated that the mutant of R231A has drastically reduced the dioxygenase activity through site-directed mutagenesis studies (Sugimoto et al., 2006). Initial structure–activity relationship (SAR) studies at the furazan 3-amino position of INCB024360 long hydrocarbon chain tail, as well as biochemical and cellular assays, proved that the introduction of a series of large and hydrophobic substituents have little improvements in clearance or inhibitory activity *in vitro*. By reducing the binding-capacity of proteins and adding polar capping groups to the amino-ethyl C3 substituent, a series of highly active IDO1 inhibitors have been obtained (Yue et al., 2017). Combined with the results of energy decomposition and hydrogen bond analysis (see sections Key Residues Scanning and Hydrogen Bond Analysis)–R231 contributes the most to the association of INCB024360 with IDO1 by forming hydrogen bonds, It is speculated that the polar group at the tail of INCB024360 stabilizes the stretched conformation of R231, thereby weakening the catalytic function of IDO1. It is a good idea to design highly active IDO1 inhibitors by adjusting its charge characteristics and then enhancing the electrostatic/hydrogen bond interactions with R231, which will prevent Trp substrate entering the active site and block the formation of hydrogen bonds between R231 and G380.

As for the inhibitor, intramolecular hydrogen bonds are helpful to promote ligand-target protein recognition, improving the stability and high affinity of the system (Wang et al., 2000). In INCB024360 and its analog INCB14943, the oxime hydroxyl group forms strong intramolecular hydrogen bonds with the furazan NH2 group and the NH group in the linkage moiety (Jorgensen et al., 1983; Yue et al., 2017). Those hydrogen bonds not only increase molecular permeability by reducing its polarity, but also stabilize the typical high-energy cis-conformation of the amidine. Besides in INCB14943 derivatives, the deletion or replacement of the hydroxyl group with a NH2 substituent resulted in a significant decrease in potency (Yue et al., 2009). According to Tojo et al., the strong basicity of nitrogen atom is conducive to the strong binding of IDO1 heme iron, which needs to be considered in the design of efficient inhibitory (Tojo et al., 2014). Lewis-Ballester et al. proposed that G262 plays a key role in substrate recognition by observing the crystal structure of IDO1-Trp (Lewis-Ballester et al., 2017). In this work (see sections Overall Dynamic Characteristics of the Trajectories and Hydrogen Bond Analysis), INCB024360's participation in the hydrogen bond network still remained stable after long-time MD simulation, in which alkaline furazan group was stabilized by L234, and G262 was recruited to form a hydrogen bond, then leading the closure of the ligand delivery tunnel and showing enhanced inhibitory activity. It has reconfirmed that the hydroxyl and furazan groups of IDO1 inhibitors are worthy of retention.

Previous studies suggested that the mutations for polar amino acids (such as S167A, C129A, and S263A) do not reduce the catalytic activity of the dioxygenase as expected (Sugimoto et al., 2006). As shown from the binding free energy calculation in section Binding Free Energy Calculation, non-polar interactions are the main driving force promoting the formation of IDO1-INCB024360. It is necessary to strengthen the interactions between inhibitors and the hydrophobic residues in IDO1's binding pockets. In addition, the hydrocarbon chain tail of INCB024360 is long and flexible. According to the perspective of Tojo et al. (2014), controlling the flexibility and length of inhibitor's tail, and introducing a linear and rigid linker both may be good strategies to increase the binding capacity with pocket B, as well as its inhibitory activity.

The formation of IDO1-INCB14943 complex mainly depends on the firm chelation of the N atom with heme iron, accompanied by the halogen-bond interactions between the chlorine atom (3-Cl) and the sulfur atom of C129 (Jorgensen et al., 1983). The chelation of O atom with heme iron and fluorine atom (4-F) with C129, both are critical to maintaining the stability of IDO1 complex with INCB024360 (Ryckaert et al., 1977). The conformation of oxime-furazan in amidoxime derivatives is relatively stable due to the two intramolecular hydrogen bonds. Thus, it is speculated that molecular recognitions of N/O-atoms and heme iron in amidoxime are very important for the development of this class of inhibitors: the latter has relatively higher inhibitory activity, while the former exhibits stronger binding ability. According to the key residues scanning result (see section Key Residues Scanning), C129 is not in the top ten residues that contribute the most to the association of INCB024360 with IDO1. In order to obtain a highly active inhibitor, it is possible to satisfy the binding of 4-fluorine atom with C129, and simultaneously modify the chain tail to improve the stability of the O-atom localization.

## Materials and Methods

All computational studies were carried out in four single PCs, running on two Intel Xeon E5-2643v3 processors with 32 GB RAM, 2 TB hard disk and Red Hat Linux Enterprise version 6.5 (Red Hat, Inc., Raleigh, NC, USA) as the operating system.

### Comparative Molecular Dynamics Simulations

Two 100 ns comparative molecular dynamics (MD) simulations were conducted for the systems of IDO1 monomer and its complex with inhibitor INCB024360. Indoleamine 2, 3-dioxygenase (IDO) includes two subtypes: IDO1 and indoleamine 2, 3-dioxygenase 2 (IDO2). Due to the lack of research on IDO2, the current IDO expression refers to IDO1 by default. The three-letter ID is an identifier for the ligand in Protein Data Bank (PDB). As for INCB024360, BBJ is its ligand ID. Thus, in this work, IDO is used to represent for the IDO1 monomer system, as well as IDO-BBJ for IDO1-INCB024360 complex. The IDO-BBJ system is based on the PDB database (ID: 5WN8), removing the crystal water and chain B, and only retaining chain A, heme and inhibitor INCB024360 (PDB code: 5WN8). The IDO system is obtained from the IDO-BBJ system in the absence of inhibitor INCB024360. In this work, all MD simulations were performed with AMBER 12 software package and AMBER force field (Wang et al., 2000). The simulation temperature was set at 300K initially and TIP3P water model was applied (Jorgensen et al., 1983). Three Na^+^ counterions were added to the two systems, with the box boundary of 10 Å.

In order to reduce the space collision of the simulated systems, two energy optimizations were carried out for IDO and IDO-BBJ, respectively. In the first energy minimization (EM) stage, the solute was constrained with force constant of 2.09 × 10^5^ kJ·mol^−1^·nm^−2^, composed of 5,000 steps with the steepest descent and 5,000 steps with the conjugate gradient. The second optimization was in the case of completely removed constraints with the steepest descent of 5,000 steps and the conjugate gradient of 5,000 steps. The energy convergence threshold of the two minimizations was <4.182 × 10^−4^ kJ·mol^−1^·nm^−2^. After EM procedure, MD simulations started, which were also divided into two stages. Firstly, solute was constrained with a force constant of 41.82 kJ·mol^−1^·nm^−2^ and the temperature increased from 0 to 300 K gradually in the initial 2.5 ns; Whereafter, a 97.5 ns unconstrained MD simulation was performed, adopting SHAKE algorithm (Ryckaert et al., 1977) to constrain the hydrogen-containing atoms with non-bonded interaction radius of 10 Å. Integration step was set as 2 fs and the atomic coordinates were stored every 1 ps, as a result, total 100,000 conformations were collected for the following structural analyses.

### Cluster Analysis

Cluster analyses of 100,000 molecular conformations obtained from MD simulations in IDO and IDO-BBJ systems were performed using the MMTSB software package (Kabsch and Sander, 1983). The basic idea of cluster analysis is to calculate the root mean square deviation (*RMSD*) values of C_α_ between various conformations and establish the *RMSD* matrix (N × N, where N is the number of conformations). First, we defined a *RMSD* threshold, if the *RMSD* between two arbitrary conformations is smaller than the threshold, they are grouped into one certain cluster and the lowest energy conformation is taken as the representative conformation. The cluster classification rules are shown in Equation 1.

(1) $$\text{C}=\{\begin{array}{c} 1\text{ for }\Delta \text{RMSD}\leq 2.4\text{ ​​}\\ 0\text{ for }\Delta \text{RMSD}>2.4\; \end{array}$$

For example, when *RMSD* is less than the threshold of 2.4 Å, C=1 indicated the two conformations are in the same cluster. Conversely, C=0 indicated they belong to the same cluster. The computational formula of *RMSD* is:

(2) $$\text{RMSD }=\sqrt{\frac{1}{N}\sum_{\text{i}=1}^{N}\delta \begin{array}{c} 2\\ i \end{array}}$$

where N is the total number of atoms and δ means the translational distance between corresponding atoms in two different conformations.

### Free Energy Landscape and Principal Component Analysis

The main idea of free energy landscape (FEL) is considering the minimum value of free energy surface (corresponding to the most stable state of system) and demarcation point (relating to a transient state in the process of system change) to investigate molecular motion and conformational changes of biological macromolecular systems. In addition, PCA is widely used to describe the most important kinetic processes in the system. Based on the MD simulation trajectory, PCA can reduce the motion of the biological macromolecule system into two categories: functional slow motion with large amplitude, whose conformation transition shows non-Gaussian distribution, and fast motion with narrow Gaussian distribution and less functional information (Fiori et al., 2015). The first principal component (PC1) and the second principal component (PC2) both serve as reaction coordinates for the mapping of free energy surface diagram (Hegger et al., 2007). The calculation of free energy is defined as:

(3) $$\Delta \text{G}(X)\;=\;-{\text{k}}_{B}\text{TlnP}(X)$$

where the reaction coordinate *X* is PC1, 2, and *k*_*B*_ is the Boltzmann constant. *T* stands for the absolute temperature in Kelvin. *P(X)* represents the contribution of a particular conformation to the overall PCs, apprehended as the probability of conformational distribution. In this work, PC1 and PC2 calculations were the basis for FEL analyses to investigate conformational changes of biological macromolecular systems.

### Binding Free Energy Calculation

Conformations were extracted from the MD trajectories of the IDO and IDO-BBJ systems every 0.5 ns intervals from 10 to 100 ns (i.e., the period when the trajectory remains in equilibrium). The average binding free energy based on the total 180 conformations was calculated with MM/PBSA method. The formula is as follows:

(4) $$\begin{array}{l} \Delta {\text{G}}_{\text{bind}}\;=\;\Delta \text{H}-\text{T}\Delta \text{S }=\;(\Delta {\text{E}}_{\text{VDW}}+\;\Delta {\text{E}}_{\text{ELE}}+\\ \;\Delta {\text{G}}_{\text{PBELE}}+\;\Delta {\text{G}}_{\text{PBSUR}})\;-\text{T}\Delta \text{S} \end{array}$$

where Δ*H* represents the total enthalpy change, *T* is the absolute temperature in Kelvin, and Δ*S* refers to the total entropy change calculated using the normal mode method (Kottalam and Case, 1990). In addition, Δ*E*_VDW_ is the non-polar fraction of the intramolecular energy under vacuum, while Δ*E*_ELE_ indicates the electrostatic section. Δ*G*_PBELE_ and Δ*G*_PBSUR_ correspond to the hydrophilic and hydrophobic parts of the solvation binding free energy, respectively.

### Key Residue Scanning

The MM/GBSA (molecular mechanics/generalized born surface area) energy decomposition (Kollman et al., 2000; Wang et al., 2001) method was used to search key residues of receptor-ligand recognition. Specifically, the MM/GBSA method divides the binding energy of each residue into the vacuum intramolecular energy calculated by molecular mechanics method, the polar solvation energies calculated by the generalized Born (GB) model (Bashford and Case, 2000; Simonson, 2001) and the non-polar solvation energies computed by linear combinations of pairwise overlaps (LCPO) model. The LCPO algorithm is based on the following: the non-polar solvation energy has a high positive correlation with the solvent accessible surface area (SASA). Afterwards, the binding energy of each residues was also decomposed into the main and side chain atoms.

## Conclusions

In this work, a series of computer-aided drug design methods were used to explore the inhibition mechanism of INCB024360. The results of comparative MD simulations and the structure superimposition of IDO with IDO-BBJ systems both revealed that three segments—pockets region, ligand delivery tunnel and the Q360-G381 loop region—have significant conformational changes after binding with INCB024360: G262 moves toward L234 and forms a steady hydrogen bond; further driving the G262-A264 loop, which is directly connected to the E-helix, closer to the heme center, and making the ligand delivery tunnel narrow and disordered. From PCA and hydrogen bond analyses, it is found that the binding of INCB024360 to IDO1 breaks the interactions between the conserved G380 and R231, then leads to the closure of the enter channel of active site. It is speculated that hydrogen bond between R231 and G380 is one of the key factors to keep the stability of Q360-G381 loop, which may be valued in drug development. This paper provides detailed atomic-level dynamics information for molecular recognition of IDO1 by INCB024360, which has important reference significance in the design of novel IDO1 inhibitors.

## Data Availability Statement

Publicly available datasets were analyzed in this study. This data can be found here: http://www.rcsb.org/.

## Author Contributions

XL, YZ, and JH: data curation. XL, WL, LL, HW, and SC: formal analysis. YZ, JH, and HS: funding acquisition. XL and YZ: investigation. XL and JH: methodology and software. XL and HS: project administration. HS: resources. XL, HD, and QL: visualization. XL: writing—original draft. JH and HS: writing—review and editing.

### Conflict of Interest

The authors declare that the research was conducted in the absence of any commercial or financial relationships that could be construed as a potential conflict of interest.

## References

[B1] Abril-Rodriguez G. Ribas A. (2017). SnapShot: immune checkpoint inhibitors. Cancer Cell 31, 848–848.e841. 10.1016/j.ccell.2017.05.010 28609660

[B2] Antonia S. J. Villegas A. Daniel D. Vicente D. Murakami S. Hui R.. (2017). Durvalumab after chemoradiotherapy in stage III non-small-cell lung cancer. N. Engl. J. Med. 377, 1919–1929. 10.1056/NEJMoa1709937 28885881

[B3] Austin C. J. Kosim-Satyaputra P. Smith J. R. Willows R. D. Jamie J. F. (2013). Mutation of cysteine residues alters the heme-binding pocket of indoleamine 2,3-dioxygenase-1. Biochem. Biophys. Res. Commun. 436, 595–600. 10.1016/j.bbrc.2013.05.119 23751345

[B4] Bashford D. Case D. A. (2000). Generalized born models of macromolecular solvation effects. Annu. Rev. Phys. Chem. 51, 129–152. 10.1146/annurev.physchem.51.1.129 11031278

[B5] Beatty G. L. O'Dwyer P. J. Clark J. Shi J. G. Bowman K. J. Scherle P. A.. (2017). First-in-Human Phase I Study of the Oral Inhibitor of Indoleamine 2,3-Dioxygenase-1 Epacadostat (INCB024360) in patients with advanced solid malignancies. Clin. Cancer Res. 23, 3269–3276. 10.1158/1078-0432.CCR-16-2272 28053021 PMC5496788

[B6] Benkert P. Biasini M. Schwede T. (2011). Toward the estimation of the absolute quality of individual protein structure models. Bioinformatics 27, 343–350. 10.1093/bioinformatics/btq662 21134891 PMC3031035

[B7] Brahmer J. Reckamp K. L. Baas P. Crino L. Eberhardt W. E. Poddubskaya E.. (2015). Nivolumab versus Docetaxel in advanced squamous-cell non-small-cell lung cancer. N. Engl. J. Med. 373, 123–135. 10.1056/NEJMoa1504627 26028407 PMC4681400

[B8] Brochez L. Chevolet I. Kruse V. (2017). The rationale of indoleamine 2,3-dioxygenase inhibition for cancer therapy. Eur. J. Cancer 76, 167–182. 10.1016/j.ejca.2017.01.011 28324751

[B9] Cady S. G. Sono M. (1991). 1-Methyl-DL-tryptophan, beta-(3-benzofuranyl)-DL-alanine (the oxygen analog of tryptophan), and beta-[3-benzo(b)thienyl]-DL-alanine (the sulfur analog of tryptophan) are competitive inhibitors for indoleamine 2,3-dioxygenase. Arch. Biochem. Biophys. 291, 326–333. 10.1016/0003-9861(91)90142-6 1952947

[B10] Chang M. Y. Smith C. DuHadaway J. B. Pyle J. R. Boulden J. Soler A. P.. (2011). Cardiac and gastrointestinal liabilities caused by deficiency in the immune modulatory enzyme indoleamine 2,3-dioxygenase. Cancer Biol. Ther. 12, 1050–1058. 10.4161/cbt.12.12.18142 22157149 PMC3335939

[B11] Chauhan N. Basran J. Efimov I. Svistunenko D. A. Seward H. E. Moody P. C.. (2008). The role of serine 167 in human indoleamine 2,3-dioxygenase: a comparison with tryptophan 2,3-dioxygenase. Biochemistry 47, 4761–4769. 10.1021/bi702405a 18370410

[B12] Colombo M. P. Piconese S. (2007). Regulatory-T-cell inhibition versus depletion: the right choice in cancer immunotherapy. Nat. Rev. Cancer 7, 880–887. 10.1038/nrc2250 17957190

[B13] Curti A. Pandolfi S. Valzasina B. Aluigi M. Isidori A. Ferri E.. (2007). Modulation of tryptophan catabolism by human leukemic cells results in the conversion of CD25- into CD25+ T regulatory cells. Blood 109, 2871–2877. 10.1182/blood-2006-07-036863 17164341

[B14] Dance A. (2017). Cancer immunotherapy comes of age. Science 355:1220 10.1126/science.355.6330.1220

[B15] Fallarino F. Grohmann U. You S. McGrath B. C. Cavener D. R. Vacca C.. (2006a). Tryptophan catabolism generates autoimmune-preventive regulatory T cells. Transpl. Immunol. 17, 58–60. 10.1016/j.trim.2006.09.017 17157218

[B16] Fallarino F. Grohmann U. You S. McGrath B. C. Cavener D. R. Vacca C.. (2006b). The combined effects of tryptophan starvation and tryptophan catabolites down-regulate T cell receptor zeta-chain and induce a regulatory phenotype in naive T cells. J. Immunol. 176, 6752–6761. 10.4049/jimmunol.176.11.6752 16709834

[B17] Feig M. Karanicolas J. Brooks C. L. III. (2004). MMTSB Tool Set: enhanced sampling and multiscale modeling methods for applications in structural biology. J. Mol. Graph. Model. 22, 377–395. 10.1016/j.jmgm.2003.12.005 15099834

[B18] Fiori A. Volpi E. Zarlenga A. Bohling G. C. (2015). Gaussian or non-Gaussian logconductivity distribution at the MADE site: what is its impact on the breakthrough curve? J. Contam. Hydrol. 179, 25–34. 10.1016/j.jconhyd.2015.05.004 26024951

[B19] Garber K. (2018). A new cancer immunotherapy suffers a setback. Science 360:588. 10.1126/science.360.6389.588 29748264

[B20] Godin-Ethier J. Hanafi L. A. Piccirillo C. A. Lapointe R. (2011). Indoleamine 2,3-dioxygenase expression in human cancers: clinical and immunologic perspectives. Clin. Cancer Res. 17, 6985–6991. 10.1158/1078-0432.CCR-11-1331 22068654

[B21] Hamid O. Bauer T. M. Spira A. I. Olszanski A. J. Patel S. P. Wasser J. S. (2017). Epacadostat plus pembrolizumab in patients with SCCHN: Preliminary phase I/II results from ECHO-202/KEYNOTE-037. J. Clin. Oncol. 35:6010 10.1200/JCO.2017.35.15_suppl.6010 PMC6225502 30265610

[B22] Hegger R. Altis A. Nguyen P. H. Stock G. (2007). How complex is the dynamics of peptide folding? Phys. Rev. Lett. 98:028102. 10.1103/PhysRevLett.98.028102 17358652

[B23] Hill M. Tanguy-Royer S. Royer P. Chauveau C. Asghar K. Tesson L.. (2007). IDO expands human CD4+CD25high regulatory T cells by promoting maturation of LPS-treated dendritic cells. Eur. J. Immunol. 37, 3054–3062. 10.1002/eji.200636704 17948274

[B24] Hu J. Hu Z. Zhang Y. Gou X. Mu Y. Wang L.. (2016). Metal binding mediated conformational change of XPA protein:a potential cytotoxic mechanism of nickel in the nucleotide excision repair. J. Mol. Model. 22:156. 10.1007/s00894-016-3017-x 27307058 PMC5327499

[B25] Hu J. P. Gong X. Q. Su J. G. Chen W. Z. Wang C. X. (2008). Study on the molecular mechanism of inhibiting HIV-1 integrase by EBR28 peptide via molecular modeling approach. Biophys. Chem. 132, 69–80. 10.1016/j.bpc.2007.09.008 18037557

[B26] Jinushi T. Shibayama Y. Kinoshita I. Oizumi S. Jinushi M. Aota T.. (2014). Low expression levels of microRNA-124-5p correlated with poor prognosis in colorectal cancer via targeting of SMC4. Cancer Med. 3, 1544–1552. 10.1002/cam4.309 25081869 PMC4298381

[B27] Jorgensen W. L. Chandrasekhar J. Madura J. D. Impey R. W. Klein M. L. (1983). Comparison of simple potential functions for simulating liquid water. J. Chem. Phys. 79, 926–935. 10.1063/1.445869

[B28] Kabsch W. Sander C. (1983). Dictionary of protein secondary structure: pattern recognition of hydrogen-bonded and geometrical features. Biopolymers 22, 2577–2637. 10.1002/bip.360221211 6667333

[B29] Katz J. B. Muller A. J. Prendergast G. C. (2008). Indoleamine 2,3-dioxygenase in T-cell tolerance and tumoral immune escape. Immunol. Rev. 222, 206–221. 10.1111/j.1600-065X.2008.00610.x 18364004

[B30] Kim J. M. Rasmussen J. P. Rudensky A. Y. (2007). Regulatory T cells prevent catastrophic autoimmunity throughout the lifespan of mice. Nat. Immunol. 8, 191–197. 10.1038/ni1428 17136045

[B31] Kollman P. A. Massova I. Reyes C. Kuhn B. Huo S. Chong L.. (2000). Calculating structures and free energies of complex molecules: combining molecular mechanics and continuum models. Acc. Chem. Res. 33, 889–897. 10.1021/ar000033j 11123888

[B32] Komiya T. Huang C. H. (2018). Updates in the Clinical Development of Epacadostat and Other Indoleamine 2,3-Dioxygenase 1 Inhibitors (IDO1) for Human Cancers. Front. Oncol. 8:423. 10.3389/fonc.2018.00423 30338242 PMC6180183

[B33] Kottalam J. Case D. A. (1990). Langevin modes of macromolecules: applications to crambin and DNA hexamers. Biopolymers 29, 1409–1421. 10.1002/bip.360291008 2361153

[B34] Kwak G. Kim D. Nam G.-H. Wang S. Y. Kim I.-S. Kim S. H.. (2017). Programmed cell death protein ligand-1 silencing with polyethylenimine-dermatan sulfate complex for dual inhibition of melanoma growth. ACS Nano 11, 10135–10146. 10.1021/acsnano.7b04717 28985469 PMC5697980

[B35] Lara P. Bauer T. M. Hamid O. Smith D. C. Gajewski T. Gangadhar T. C. (2017). Epacadostat plus pembrolizumab in patients with advanced RCC: preliminary phase I/II results from ECHO-202/KEYNOTE-037. J. Clin. Oncol. 35, 4515 10.1200/JCO.2017.35.15_suppl.4515 PMC6225502 30265610

[B36] Lewis-Ballester A. Karkashon S. Batabyal D. Poulos T. L. Yeh S. R. (2018). Inhibition Mechanisms of Human Indoleamine 2,3 Dioxygenase 1. J. Am. Chem. Soc. 140, 8518–8525. 10.1021/jacs.8b03691 29897749 PMC6434940

[B37] Lewis-Ballester A. Pham K. N. Batabyal D. Karkashon S. Bonanno J. B. Poulos T. L.. (2017). Structural insights into substrate and inhibitor binding sites in human indoleamine 2,3-dioxygenase 1. Nat. Commun. 8, 1693–1693. 10.1038/s41467-017-01725-8 29167421 PMC5700043

[B38] Li M. Bolduc A. R. Hoda M. N. Gamble D. N. Dolisca S. B. Bolduc A. K.. (2014). The indoleamine 2,3-dioxygenase pathway controls complement-dependent enhancement of chemo-radiation therapy against murine glioblastoma. J. Immunother Cancer 2:21. 10.1186/2051-1426-2-21 25054064 PMC4105871

[B39] Lin S. Y. Yeh T. K. Song J. S. Hung M. S. Cheng M. F. Liao F. Y.. (2018). 4-Bromophenylhydrazinyl benzenesulfonylphenylureas as indoleamine 2,3-dioxygenase inhibitors with *in vivo* target inhibition and anti-tumor efficacy. Bioorg. Chem. 77, 600–607. 10.1016/j.bioorg.2018.02.010 29494816

[B40] Liu X. Shin N. Koblish H. K. Yang G. Wang Q. Wang K.. (2010). Selective inhibition of IDO1 effectively regulates mediators of antitumor immunity. Blood 115, 3520–3530. 10.1182/blood-2009-09-246124 20197554

[B41] Ma C. R. Liu X. X. Yu S. Zhao S. Cook J. M. (1999). Concise synthesis of optically active ring-A substituted tryptophans. Tetrahedron Lett. 40, 657–660. 10.1016/S0040-4039(98)02497-6

[B42] Macchiarulo A. Nuti R. Bellocchi D. Camaioni E. Pellicciari R. (2007). Molecular docking and spatial coarse graining simulations as tools to investigate substrate recognition, enhancer binding and conformational transitions in indoleamine-2,3-dioxygenase (IDO). Biochim. Biophys. Acta 1774, 1058–1068. 10.1016/j.bbapap.2007.06.007 17644054

[B43] Madeira F. Park Y. M. Lee J. Buso N. Gur T. Madhusoodanan N.. (2019). The EMBL-EBI search and sequence analysis tools APIs in 2019. Nucleic Acids Res. 47, W636–W641. 10.1093/nar/gkz268 30976793 PMC6602479

[B44] McGuire S. (2016). $$World Cancer Report 2014. Geneva, Switzerland: World Health Organization, International Agency for Research on Cancer, WHO Press, 2015. Adv Nutr 7, 418–419. 10.3945/an.116.012211 26980827 PMC4785485

[B45] Myint A. M. Kim Y. K. (2014). Network beyond IDO in psychiatric disorders: revisiting neurodegeneration hypothesis. Prog. Neuropsychopharmacol. Biol. Psychiatry 48, 304–313. 10.1016/j.pnpbp.2013.08.008 24184687

[B46] Nagato T. Celis E. (2014). A novel combinatorial cancer immunotherapy: poly-IC and blockade of the PD-1/PD-L1 pathway. Oncoimmunology 3:e28440. 10.4161/onci.28440 25050210 PMC4063146

[B47] Nienhaus K. Nickel E. Nienhaus G. U. (2017). Substrate binding in human indoleamine 2,3-dioxygenase 1: a spectroscopic analysis. Biochim. Biophys. Acta Proteins Proteom 1865, 453–463. 10.1016/j.bbapap.2017.02.008 28189796

[B48] Pan L. Zheng Q. Chen Y. Yang R. Yang Y. Li Z.. (2018). Design, synthesis and biological evaluation of novel naphthoquinone derivatives as IDO1 inhibitors. Eur. J. Med. Chem. 157, 423–436. 10.1016/j.ejmech.2018.08.013 30103191

[B49] Pardoll D. M. (2012). The blockade of immune checkpoints in cancer immunotherapy. Nat. Rev. Cancer 12, 252–264. 10.1038/nrc3239 22437870 PMC4856023

[B50] Peng Y. H. Ueng S. H. Tseng C. T. Hung M. S. Song J. S. Wu J. S.. (2016). Important Hydrogen Bond Networks in Indoleamine 2,3-Dioxygenase 1 (IDO1) inhibitor design revealed by crystal structures of imidazoleisoindole derivatives with IDO1. J. Med. Chem. 59, 282–293. 10.1021/acs.jmedchem.5b01390 26642377

[B51] Perez R. P. Riese M. J. Lewis K. D. Saleh M. N. Daud A. Berlin J. (2017). Epacadostat plus nivolumab in patients with advanced solid tumors: preliminary phase I/II results of ECHO-204. J. Clin. Oncol. 35, 3003 10.1200/JCO.2017.35.15_suppl.3003

[B52] Prendergast G. C. (2008). Immune escape as a fundamental trait of cancer: focus on IDO. Oncogene 27, 3889–3900. 10.1038/onc.2008.35 18317452

[B53] Reck M. Rodriguez-Abreu D. Robinson A. G. Hui R. Csoszi T. Fulop A.. (2016). Pembrolizumab versus chemotherapy for PD-L1-positive non-small-cell lung cancer. N. Engl. J. Med. 375, 1823–1833. 10.1056/NEJMoa1606774 27718847

[B54] Rittmeyer A. Barlesi F. Waterkamp D. Park K. Ciardiello F. von Pawel J.. (2017). Atezolizumab versus docetaxel in patients with previously treated non-small-cell lung cancer (OAK): a phase 3, open-label, multicentre randomised controlled trial. Lancet 389, 255–265. 10.1016/S0140-6736(16)32517-X 27979383 PMC6886121

[B55] Rohrig U. F. Majjigapu S. R. Vogel P. Zoete V. Michiein O. (2015). Challenges in the Discovery of Indoleamine 2,3-Dioxygenase 1 (IDO1) inhibitors. J. Med. Chem. 58, 9421–9437. 10.1021/acs.jmedchem.5b00326 25970480

[B56] Ryckaert J.-P. Ciccotti G. Berendsen H. J. (1977). Numerical integration of the cartesian equations of motion of a system with constraints: molecular dynamics of n-alkanes. J. Comput. Phys. 23, 327–341. 10.1016/0021-9991(77)90098-5

[B57] Schalper K. A. (2014). PD-L1 expression and tumor-infiltrating lymphocytes: revisiting the antitumor immune response potential in breast cancer. Oncoimmunology 3:e29288. 10.4161/onci.29288 25083339 PMC4106164

[B58] Shi J. G. Chen X. J. Punwani N. G. Williams W. V. Yeleswaram S. (2016). Potential Underprediction of warfarin drug interaction from conventional interaction studies and risk mitigation: a case study with epacadostat, an IDO1 Inhibitor. J. Clin. Pharmacol. 56, 1344–1354. 10.1002/jcph.737 26990117

[B59] Simonson T. (2001). Macromolecular electrostatics: continuum models and their growing pains. Curr. Opin. Struct. Biol. 11, 243–252. 10.1016/S0959-440X(00)00197-4 11297935

[B60] Smart O. S. Neduvelil J. G. Wang X. Wallace B. A. Sansom M. S. (1996). HOLE: a program for the analysis of the pore dimensions of ion channel structural models. J. Mol. Graph. 14, 354–376. 10.1016/S0263-7855(97)00009-X 9195488

[B61] Smith D. C. Gajewski T. Mid O. H. Wasser J. S. Olszanski A. J. Patel S. P. (2017). Epacadostat plus pembrolizumab in patients with advanced urothelial carcinoma: preliminary phase I/II results of ECHO-202/KEYNOTE-037. J. Clin. Oncol. 35, 4503 10.1200/JCO.2017.35.15_suppl.4503 PMC6225502 30265610

[B62] Spranger S. Koblish H. K. Horton B. Scherle P. A. Newton R. Gajewski T. F. (2014). Mechanism of tumor rejection with doublets of CTLA-4, PD-1/PD-L1, or IDO blockade involves restored IL-2 production and proliferation of CD8(+) T cells directly within the tumor microenvironment. J. Immunother Cancer 2:3. 10.1186/2051-1426-2-3 24829760 PMC4019906

[B63] Sugimoto H. Oda S. Otsuki T. Hino T. Yoshida T. Shiro Y. (2006). Crystal structure of human indoleamine 2,3-dioxygenase: catalytic mechanism of O2 incorporation by a heme-containing dioxygenase. Proc. Natl. Acad. Sci. U.S.A. 103, 2611–2616. 10.1073/pnas.0508996103 16477023 PMC1413787

[B64] Sun X. Yan X. Zhuo W. Gu J. Zuo K. Liu W.. (2018). PD-L1 nanobody competitively inhibits the formation of the PD-1/PD-L1 complex: comparative molecular dynamics simulations. Int. J. Mol. Sci. 19:E1984. 10.3390/ijms19071984 29986511 PMC6073277

[B65] Tang J. Shalabi A. Hubbard-Lucey V. M. (2018). Comprehensive analysis of the clinical immuno-oncology landscape. Ann. Oncol. 29, 84–91. 10.1093/annonc/mdx755 29228097

[B66] Thorson J. S. Chapman E. Schultz P. G. (1995). Analysis of hydrogen bonding strengths in proteins using unnatural amino acids. J. Am. Chem. Soc. 117, 9361–9362. 10.1021/ja00141a032

[B67] Tojo S. Kohno T. Tanaka T. Kamioka S. Ota Y. Ishii T.. (2014). Crystal structures and structure-activity relationships of imidazothiazole derivatives as IDO1 inhibitors. ACS Med. Chem. Lett. 5, 1119–1123. 10.1021/ml500247w 25313323 PMC4190630

[B68] Tomek P. Palmer B. D. Flanagan J. U. Sun C. Raven E. L. Ching L. M. (2017). Discovery and evaluation of inhibitors to the immunosuppressive enzyme indoleamine 2,3-dioxygenase 1 (IDO1): probing the active site-inhibitor interactions. Eur. J. Med. Chem. 126, 983–996. 10.1016/j.ejmech.2016.12.029 28011425

[B69] Torre L. A. Bray F. Siegel R. L. Ferlay J. Lortet-Tieulent J. Jemal A. (2015). Global cancer statistics, 2012. CA Cancer J. Clin. 65, 87–108. 10.3322/caac.21262 25651787

[B70] Ul Haq F. Abro A. Raza S. Liedl K. R. Azam S. S. (2017). Molecular dynamics simulation studies of novel β-lactamase inhibitor. J. Mol. Graph. Model. 74, 143–152. 10.1016/j.jmgm.2017.03.002 28432959

[B71] van der Goot A. T. Zhu W. Vázquez-Manrique R. P. Seinstra R. I. Dettmer K. Michels H.. (2012). Delaying aging and the aging-associated decline in protein homeostasis by inhibition of tryptophan degradation. Proc. Natl. Acad. Sci. U.S.A. 109, 14912–14917. 10.1073/pnas.1203083109 22927396 PMC3443121

[B72] Wang J. Cieplak P. Kollman P. A. (2000). How well does a restrained electrostatic potential (RESP) model perform in calculating conformational energies of organic and biological molecules? J.Comput. Chem. 21, 1049–1074. 10.1002/1096-987X(200009)21:12&lt;1049::AID-JCC3&gt;3.0.CO;2-F

[B73] Wang W. Donini O. Reyes C. M. Kollman P. A. (2001). Biomolecular simulations: recent developments in force fields, simulations of enzyme catalysis, protein-ligand, protein-protein, and protein-nucleic acid noncovalent interactions. Annu. Rev. Biophys. Biomol. Struct. 30, 211–243. 10.1146/annurev.biophys.30.1.211 11340059

[B74] Xie Q. Wang L. Zhu B. Wang Y. Gu J. Chen Z. (2008). The expression and significance of indoleamine−2,3-dioxygenase in non-small cell lung cancer cell. Zhongguo Fei Ai Za Zhi 11, 115–119. 10.3779/j.issn.1009-3419.2008.01.025 20727279

[B75] Xie T. Wu Z. Gu J. Guo R. Yan X. Duan H.. (2019). The global motion affecting electron transfer in *Plasmodium falciparum* type II NADH dehydrogenases: a novel non-competitive mechanism for quinoline ketone derivative inhibitors. Phys. Chem. Chem. Phys. 21, 18105–18118. 10.1039/C9CP02645B 31396604

[B76] Yamazaki F. Kuroiwa T. Takikawa O. Kido R. (1985). Human indolylamine 2,3-dioxygenase. Its tissue distribution, and characterization of the placental enzyme. Biochem. J. 230, 635–638. 10.1042/bj2300635 3877502 PMC1152665

[B77] Yan D. J. Lin Y. W. Tan X. S. (2017). Heme-containing enzymes and inhibitors for tryptophan metabolism. Metallomics 9, 1230–1240. 10.1039/C7MT00105C 28650043

[B78] Yang S. S. Li X. S. Hu F. F. Li Y. L. Yang Y. Y. Yan J. K.. (2013). Discovery of Tryptanthrin Derivatives as Potent Inhibitors of Indoleamine 2,3-Dioxygenase with Therapeutic Activity in Lewis Lung Cancer (LLC) Tumor-Bearing Mice. J. Med. Chem. 56, 8321–8331. 10.1021/jm401195n 24099220

[B79] Yue E. W. Douty B. Wayland B. Bower M. Liu X. D. Leffet L.. (2009). Discovery of potent competitive inhibitors of indoleamine 2,3-Dioxygenase with *in vivo* pharmacodynamic activity and efficacy in a mouse melanoma model. J. Med. Chem. 52, 7364–7367. 10.1021/jm900518f 19507862

[B80] Yue E. W. Sparks R. Polam P. Modi D. Douty B. Wayland B.. (2017). INCB24360 (Epacadostat), a highly potent and selective indoleamine-2,3-dioxygenase 1 (IDO1) inhibitor for immuno-oncology. ACS Med. Chem. Lett. 8, 486–491. 10.1021/acsmedchemlett.6b00391 28523098 PMC5430407

[B81] Zamanakou M. Germenis A. E. Karanikas V. (2007). Tumor immune escape mediated by indoleamine 2,3-dioxygenase. Immunol. Lett. 111, 69–75. 10.1016/j.imlet.2007.06.001 17644189

[B82] Zeeberg Iversen T. Andersen M. Svane I. (2014). The targeting of IDO-mediated immune escape in cancer. Basic Clin. Pharmacol. Toxicol. 116, 19–24. 10.1111/bcpt.12320 25207460

[B83] Zhai L. Spranger S. Binder D. C. Gritsina G. Lauing K. L. Giles F. J.. (2015). Molecular pathways: targeting IDO1 and other tryptophan dioxygenases for cancer immunotherapy. Clin. Cancer Res. 21, 5427–5433. 10.1158/1078-0432.CCR-15-0420 26519060 PMC4681601

[B84] Zheng X. Koropatnick J. Li M. Zhang X. Ling F. Ren X.. (2006). Reinstalling antitumor immunity by inhibiting tumor-derived immunosuppressive molecule IDO through RNA interference. J. Immunol. 177, 5639–5646. 10.4049/jimmunol.177.8.5639 17015752

