# Supplementary material for: Inhibition Mechanism of Indoleamine 2, 3-Dioxygenase 1 (IDO1) by Amidoxime Derivatives and Its Revelation in Drug Design: Comparative Molecular Dynamics Simulations
Source: Front Mol Biosci. 2020 Jan 28;6:164. doi: 10.3389/fmolb.2019.00164 (PMC6997135; doi:10.3389/fmolb.2019.00164)
Supplement: Supplementary file 1 [file Data_Sheet_1.docx]

Supplementary Material

**Table S1.** Bioinformatics data for IDO1 structures currently available.

| **PDB ID** | **Resolution（**Å**）** | | **Organism（s）** | **Small Molecules** | | **Chains** | **Mutation（s）** |
| --- | --- | --- | --- | --- | --- | --- | --- |
| 5WHR | 2.28 | Homo sapiens | | AOJ, HEM | A, B | | 0 |
| 2D0T | 2.3 | Homo sapiens | | HEM, NHE, PIM | A, B | | 0 |
| 2D0U | 3.4 | Homo sapiens | | CYN, HEM, NHE | A, B | | 0 |
| 4PK5 | 2.79 | Homo sapiens | | HEM, PKJ | A, B | | 0 |
| 4PK6 | 3.45 | Homo sapiens | | HEM, PKL | A, B | | 0 |
| 4U72 | 2.0 | Homo sapiens | | HEM, NHE, PIM | A, B | | 1 |
| 4U74 | 2.31 | Homo sapiens | | HEM, NHE, PIM | A, B | | 1 |
| 5EK2 | 2.68 | Homo sapiens | | 5PJ, HEM | A, B | | 0 |
| 5EK3 | 2.21 | Homo sapiens | | 5PK, HEM | A, B | | 0 |
| 5EK4 | 2.64 | Homo sapiens | | 5PF, HEM | A, B | | 0 |
| 5ETW | 2.7 | Homo sapiens | | HEM, XNL | A, B | | 0 |
| 5WMU | 2.4 | Homo sapiens | | CYN, HEM, TRP | A, B | | 0 |
| 5WMV | 2.6 | Homo sapiens | | CYN, HEM, TRP, ZCW | A, B | | 0 |
| 5WMW | 3.03 | Homo sapiens | | CYN, HEM, TRP | A, B | | 1 |
| 5WMX | 2.69 | Homo sapiens | | CYN, HEM, TRP, ZCW | A, B | | 1 |
| 5WN8 | 2.5 | Homo sapiens | | BBJ, HEM | A, B | | 0 |
| 5XE1 | 3.2 | Homo sapiens | | HEM, IUU | A, B | | 0 |
| 6AZU | 2.82 | Homo sapiens | | HEM, SO4 | A, B, C, D | | 0 |
| 6AZV | 2.76 | Homo sapiens | | C4V | A, B, C, D | | 0 |
| 6AZW | 2.78 | Homo sapiens | | C51 | A, B | | 0 |
| 6CXU | 2.49 | Homo sapiens | | CYN, HEM, TRP | A, B | | 1 |
| 6CXV | 2.6 | Homo sapiens | | CYN, HEM, TRP, ZCW | A, B | | 1 |
| 6E35 | 2.41 | Homo sapiens | | CYN, HEM, TRP, ZCW | A, B | | 0 |
| 6F0A  6O3I  6DPQ  6DPR  6MQ6  6E40  6E41  6E42  6E43  6E44  6E45  6E46 | 2.26  2.69  2.94  3.2  3.05  2.31  2.29  2.1  1.71  1.9  2.0  2.09 | Homo sapiens  Homo sapiens  Homo sapiens  Homo sapiens  Homo sapiens  Homo sapiens  Homo sapiens  Homo sapiens  Homo sapiens  Homo sapiens  Homo sapiens Homo sapiens | | ALA, C82, HEM  LKP, HEM  GOL, H7P, HEM  EDO, H7P, HEM  GOL, H7P, HEM  BBJ, HEM  HEM, HQS  HEM, HQJ, PO4  BEZ, HQM  HEM, PO4  GOL, HEM, PO4  HEM, PO4, TRP | A, C  A, B  A, B  A, B  A, B  A, B, C, D  A, B, C, D  A, B, C, D  A, B, C, D  A, B, C, D  A, B, C, D  A, B, C, D | | 0  0  0  0  0  2  2  2  2  2  2  2 |


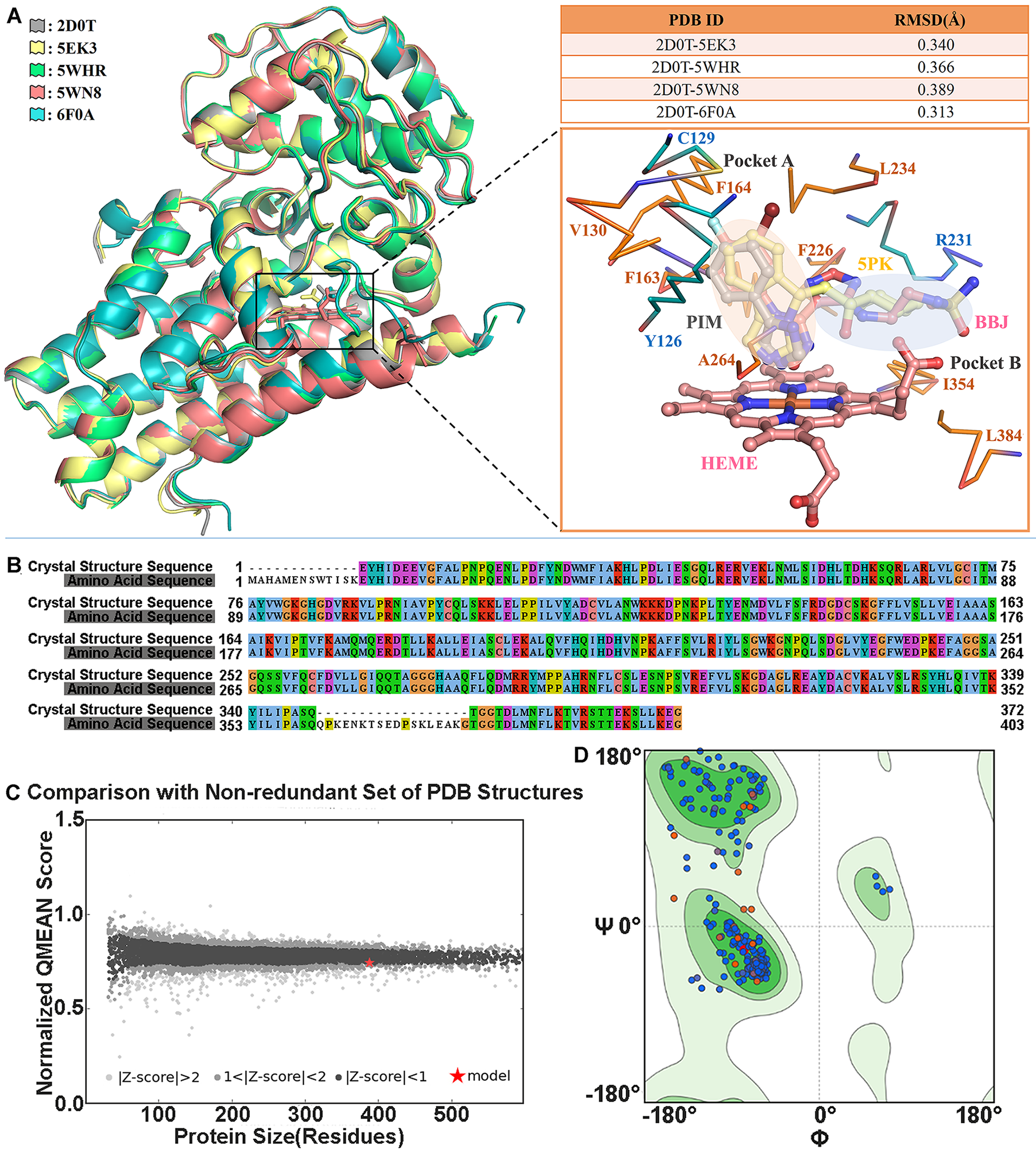


**Figure S1.** Screening results of the research systems, sequence alignment and modeling quality evaluations of 5WN8. Amino acids that make up pockets are marked with color orange and blue, depending on the polarity. Inhibitors are named in accordance with that in crystal structures. PIM, 5PK and BBJ refer to 4-phenylimidazole, NLG919 analogue and INCB024360, respectively (**A**). Sequence alignment between IDO1 primary amino acid sequence and that of 5WN8 (**B**). QMEAN Z-score evaluation of IDO1. The x-axis shows protein length (number of residues). The y-axis is the normalized QMEAN score. Every dot represents one experimental protein structure. Black, gray and light gray dots stand for the experimental structures with a normalized QMEAN score (i.e., |Z-score|) of less than 1, between 1 and 2, and greater than 2, respectively. Experimental structure that are even further from the mean are light grey. The actual model is represented as a red star. The mean and standard deviation of the experimental structures around the x-location of the star are the basis to calculate the QMEAN Z-score of the model (**C**). Ramachandran plot analysis of IDO1. Color green in center indicates the conformational optimal region illustrating that the more amino acids appear in this region, the more reliable the structure established. The light green region represents the conformational allowed area and the remainder was the forbidden one (**D**).


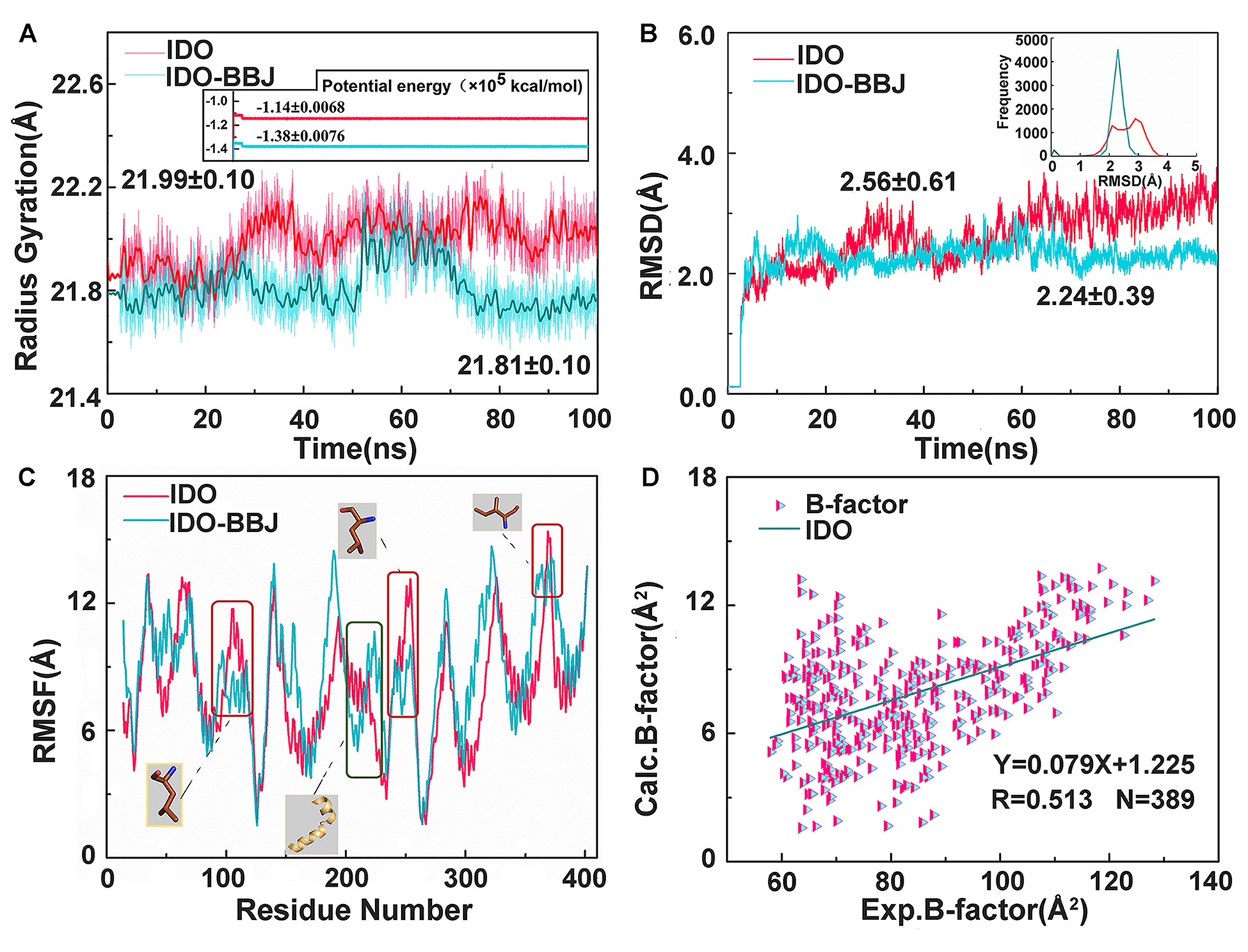


**Figure S2.** Molecular dynamics analyses of IDO and IDO-BBJ systems. Potential energy and radius gyration analyses of IDO (in red) and IDO-BBJ (in blue) systems (**A**); the analyses of RMSD and its frequency distribution (**B**); RMSF distribution of Cα atoms, where single residues (L103, L234, I354) and pocket region (L200-R231) are described as stick and cartoon modes, respectively (**C**); correlation of calculated and experimental B-factor values (**D**).

**Table S2.** Prediction of binding free energy in the IDO-Trp and IDO-BBJ systems (kJ·mol^−1^).

| **Systems** | **Items** | **IDO** | | **Ligand** | **Complex** | | **Delta** |
| --- | --- | --- | --- | --- | --- | --- | --- |
|  | ***ELE*_IN_** | -11510.87±175.46 | | -385.65±10.05 | -11946.40±178.47 | | -49.88±6.64 |
|  | ***VDW*_IN_** | -1831.33±27.45 | | 9.08±2.93 | -1873.53±26.20 | | -51.28±2.22 |
|  | ***ELE*_PB_** | -4009.87±156.78 | | -31.37±2.18 | -3975.44±157.34 | | 65.81±3.43 |
| **IDO-BBJ** | ***VDW*_PB_** | 98.58±1.83 | | 4.04±0.03 | 97.50±1.84 | | -5.12±0.05 |
|  | ***H*** | -7803.92±71.67 | | -266.56±2.97 | -8110.87±72.22 | | -40.47±3.92 |
|  | ***T*Δ*S*** | 4363.04±8.31 | | 53.80±0.45 | 4397.38±9.34 | | -19.46±3.99 |
|  | **Δ*G*_bind_** | -21.01 | | | | | |
|  | ***ELE*_IN_** | -11603.18±260.44 | 8.67±3.42 | | -11620.86±256.20 | -26.35±7.56 | |
|  | ***VDW*_IN_** | -1884.99±60.07 | 3.05±0.70 | | -1918.61±59.26 | -36.67±2.66 | |
|  | ***ELE*_PB_** | 91.84±1.20 | -3.12±0.02 | | 91.20±1.23 | -3.76±0.07 | |
| **IDO-Trp** | ***VDW*_PB_** | -3606.43±122.09 | -22.04±2.18 | | -3586.64±120.28 | 41.83±4.75 | |
|  | ***H*** | -8028.71±458.69 | 30.90±4.55 | | -8022.76±455.41 | -24.95±4.05 | |
|  | ***T*Δ*S*** | 4267.71±6.51 | 35.79±0.20 | | 4293.10±9.90 | -10.40±6.74 | |
|  | **Δ*G*_bind_** | -14.55 | | | | | |
